# The psychometric properties of GHQ for detecting common mental disorder among community dwelling men in Goa, India

**DOI:** 10.1016/j.ajp.2017.03.023

**Published:** 2017-08

**Authors:** Paige Endsley, Benedict Weobong, Abhijit Nadkarni

**Affiliations:** a Columbia University, Mailman School of Public Health, United States; b Sangath, 841/1, Alto Porvorim, Bardez, B/H Electricty Dept, H No 451 (168), Bhatkar Waddo, Socorro, Bardez, Porvorim, Goa 403521, India; c London School of Hygiene and Tropical Medicine, Keppel Street, London WC1E 7HT, UK

**Keywords:** Common mental disorders, GHQ, Validation, Goa, India

## Abstract

**Background:**

There have not been many attempts to validate screening measures for common mental disorders (CMD) in low- and middle-income countries. The aim of this study was to examine the criterion validity of the General Health Questionnaire 12 (GHQ-12) in a community-based study from Goa, India.

**Method:**

Concurrent and convergent validity of the GHQ-12 were assessed against the Mini International Neuropsychiatric Interview (MINI) and World Health Organization Disability Assessment Scale (WHODAS) for CMD and functional status through the secondary analysis of a community cohort of men from Goa, India. Criterion validity of the GHQ-12 was determined using ROC analyses with the MINI case criterion as the gold standard. Concurrent validity was assessed against the gold standard of WHODAS functional disability and number of disability days.

**Results:**

In a sample of men (*n* = 773), the GHQ-12 showed high internal reliability (Cronbach's alpha of 0.82) and acceptable criterion validity (Area under the receiver operating characteristic curve being 0.71). It had adequate psychometric properties for the detection of CMD (sensitivity of 68.75%; specificity of 73.14%) with the optimal cut-off score for identification of CMD being 2.

**Conclusion:**

In order to optimize the usefulness and validity of the GHQ-12, a low cut-off point for CMD may be beneficial in Goa, India. Further validation studies for the GHQ-12 should be conducted for continued validation of the test for use in the community.

## Highlights

• GHQ-12 is fairly effective, efficient screening tool for CMD in Goa, India.
• Optimal cut-off score was found to be lower than WHO-recommended score.
• Lowered cut-off point is recommended in Goa, India for community use.
• Cut-off point may have implications for CMD identification in community.

## 1 Introduction

### 1.1 The burden of common mental disorders

Common mental disorders (CMDs) are defined as depressive non-psychotic symptoms, anxiety and somatic complaints that affect the performance of daily activities, incorporating depressive and anxiety disorders (Goldberg and Huxley, 1992). The WHO World Mental Health surveys estimate global lifetime prevalence of all mental disorders between 18.1 and 36.1% (Kessler et al., 2009) and that of CMDs is between 25.9 and 32.6% (Steel et al., 2014). The Global Burden of Disease survey found mental health and substance use disorders to account for the majority of years lived with disability (YLD), with depressive and anxiety disorders comprising over half of those YLDs (Whiteford et al., 2013).

### 1.2 The importance of GHQ-12 as a screening tool

Despite the large variety of screening tools available for identifying CMD very few have been specifically designed for LMIC populations. The concern for cross-cultural psychiatry is that most of these tools developed in high-income settings will miss cases in LMIC settings, the epidemiology and clinical presentations of mental health problems differ between settings (Kirmayer, 2001). For example, previous validations for CMD measures, including the GHQ-12, have found lower optimal cut-off scores than those recommended for the populations in which the tools were originally developed (Kim et al., 2013, Adewuya et al., 2006).

The GHQ-12 is particularly recommended for assessing CMD (Goldberg, 1979), because it has the strongest psychometric properties among other tools (Goldberg et al., 1997, Schmitz et al., 1999). It is especially attractive in primary care settings where efficiency and brevity are valued. It was adopted in a WHO study screening for psychological disorders in primary care and has been deemed the most valid among similar tools (Goldberg et al., 1997, Schmitz et al., 1999), albeit in settings with similar characteristics as where it was originally developed. Its validation for CMDs in LMIC is seriously underrepresented and a recent systematic review identified only 13 validation studies in LMICs (Ali et al., 2016). Continued validation of the GMQ-12 in LMICs is thus warranted and in line with the recommendation that a chosen tool should be validated in the context in which it will be employed (Ali et al., 2016).

While the GHQ-12 has been used in Goa, its validity and usefulness has only been established for use in primary-care settings, not within the community (Pillai et al., 2013, Patel and Prince, 2006, Patel et al., 2008); validity studies from high prevalence clinical settings may not generalize to the community as the process of seeking healthcare, the interaction with clinicians, and relatively high proportions of more severe disorders may all lead to bias (Carey et al., 2003). In this report, we describe the validity and reliability of the Konkani (local language of Goa) version of the GHQ-12 among a community-based sample in Goa, India.

## 2 Methods

### 2.1 Setting

This sub-study is a part of a large community-based cohort study conducted in Goa, which has a population of just over 1.4 million, 62% of whom live in urban areas (Chandramouli and India, 2011).

### 2.2 Participants and follow up procedures

Participants were adult males aged 18–49 years (at baseline) first interviewed between 2006 and 2008, and then completed a follow-up survey 6–8 years later. Study sites included urban (two beach areas popular among tourists and one typical commercial and residential area) and rural areas (six contiguous villages) of Northern Goa (Pillai et al., 2013). At baseline, a two-stage probability sampling procedure, based on electoral rolls, was employed to determine the population-based sample. The participants were selected at random from those with eligible ages within the randomly selected households. Refusal rate for randomly selected households was 1.5%.

At a follow-up from September 2012 to September 2014, a range of self-reported outcomes were measured on the baseline cohort, including GHQ-12, MINI, and WHODAS. All consenting participants were administered the self-report questionnaire by trained research workers. The research workers were blind to the study hypothesis, and CMD status at baseline. The data analyzed and presented here were taken only from the follow-up measurements. Quality control was conducted by re-interviewing 10% randomly selected participants by the research coordinator and random visits by the research coordinator to directly observe the research workers.

### 2.3 Ethics

Ethical approval was obtained from the Sangath Institutional Review Board, ethics committee of the London School of Hygiene and Tropical Medicine and the Indian Council of Medical Research. Each research worker completed the NIH Protecting Human Research Participant online course. Participants diagnosed with AUD or CMD were offered further free clinical assessment and treatment by a psychiatrist.

### 2.4 Assessments

#### 2.4.1 Gold standard criterion measure

##### 2.4.1.1 MINI

The Mini International Neuropsychiatric Interview (MINI) was used to identify current common mental disorders (Lecrubier et al., 1997). The MINI is a short diagnostic structured interview to explore 17 disorders according to Diagnostic and Statistical Manual IV-TR diagnostic criteria. It allows for administration by non-specialized interviewers. Interviews were conducted using paper and pencil with diagnosis assessed following a structured algorithm.

#### 2.4.2 Concurrent validity measure

##### 2.4.2.1 WHODAS

The WHO Disability Assessment Schedule (WHODAS) is a 12-item questionnaire for measuring functional impairment over the previous 30 days. In addition, two items assess number of days the person was unable to work in the previous 30 days. The WHODAS has uniform response options ranging from 0 to 4, and provides a continuously distributed summed up score of up to 48. In the present analyses, the WHODAS was used to assess health and general disability and functional status of participants. The WHODAS assesses disability in a range of functions including: standing, walking, concentrating, learning, household responsibilities, maintaining personal hygiene, dressing, social relationships, work, and emotions due to health problems.

#### 2.4.3 Test measure

##### 2.4.3.1 General Health Questionnaire-12 (GHQ-12)

The GHQ-12 was used to screen for CMD. The questionnaire asked whether the respondent had experienced a particular symptom or behavior recently, and each item was rated on a set of four response options (less than usual, no more than usual, rather more than usual, or much more than usual). Scoring of the GHQ-12 was done in the original bi-modal method as developed by Goldberg (1979). Thus, based on the response options, items were scored as 0, 0, 1, or 1 respectively. This scoring method allowed for total scores to range from 0 to 12.

### 2.5 Statistical methods

The psychometric properties of the GHQ-12 were determined using Receiver Operating Characteristics (ROC) analysis with the MINI case criterion as the gold standard in order to generate the area under the curve and the optimal cut-point. The ROC analysis also yielded sensitivity and specificity estimates, including likelihood ratios (+/−) at that cut-point. In addition to this, we estimated Youden's index, a measure of overall test performance (sensitivity + specificity – 1), in order to compare our validity coefficients directly with those reported in other similar studies (Fluss et al., 2005). To further compare our results to others, diagnostic odds ratio (DOR) was also computed as a measure of screening tool effectiveness. Agreement between the test cut-point and the gold standard was assessed using Cohen's Kappa. The internal scale consistency of the measure was ascertained by Cronbach's alpha. Concurrent validity of the GHQ-12 was assessed with Pearson's correlation coefficient for the correlation with the WHODAS functional disability and number of disability days. An item-level analysis was then conducted to determine if there were item-level difficulties in detecting CMD case. The item-level analysis included Pearson's item-total correlation, Cronbach's alpha coefficient if each item is removed, and the likelihood positive ratio. All analyses were conducted using STATA 13.

## 3 Results

Seven hundred and seventy-three men completed both the GHQ and the MINI. Mean age was 33.2 years at the baseline survey (range 18–49, SD 8.44). According to the MINI gold-standard criterion 32/773 (4.1%) had CMD. The prevalence by the GHQ-12 was 39/773 (5.1%), when using the recommended cut-off score of 6. The mean score for GHQ among the sample was 1.36 (SD = 2.17). The median was 0 with an interquartile range of 0–2 (Table 1).

**Table 1:** Test scale distribution, reliability, and validity.

Test scale	GHQ
*Scale distribution*	
Mean	1.36 (sd = 2.17)
Median (IQR)	0 (0–2)

*Scale reliability*	
Cronbach's alpha	0.82
Kappa	0.11

*Criterion validity against MINI gold standard*	
AUROC	0.71
Optimal cut-point	2

*At this cut-point*	
Sensitivity	68.8%
Specificity	73.1%
LR+	2.56
LR−	0.43
Youden's Index	0.38
Prevalence	28.6% (221/773)* using cut-off of 2

*Concurrent validity*	
WHODAS disability	0.23
Disability days	0.18

**Fig. 1:**
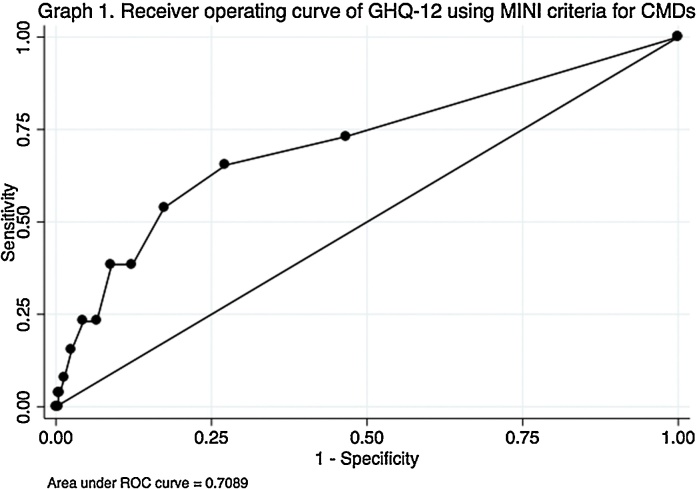
Receiver operating curve of GHQ-12 using MINI criteria for CMDs.

The area under the ROC (Fig. 1) for the GHQ was found to be 0.71, when using the MINI as the gold standard. Youden's index of 0.38 pointed to the optimal cut-off point of 2 for identifying CMD among the sample using the GHQ. At this cut-off point of 2, sensitivity and specificity against the MINI were found to be 68.75% and 73.14% respectively, and the prevalence of CMD was 28.59%. A detailed summary of psychometric properties at each cut-off point is presented in Table 2. Diagnostic odds ratios were computed to be 5.95 for the cut-off of 2 and 4.93 for the cut-off of 6, both of which indicate weak diagnostic properties.

**Table 2:** Psychometric properties of GHQ-12 at different cut-off points.

Cutpoint	Sensitivity	Specificity	Correctly classified	LR+	LR−	DOR
≥0	100.00%	0.00%	4.14%	1		
≥1	75.00%	53.71%	54.59%	1.62	0.47	
≥2	68.75%	73.14%	72.96%	2.56	0.43	5.95
≥3	56.25%	83.00%	81.89%	3.31	0.53	
≥4	43.75%	88.12%	86.29%	3.68	0.64	
≥5	37.50%	91.23%	89.00%	4.28	0.69	
≥6	25.00%	93.66%	90.82%	3.94	0.8	4.93
≥7	25.00%	95.82%	92.88%	5.98	0.78	
≥8	12.50%	97.44%	93.92%	4.88	0.9	
≥9	6.25%	98.65%	94.83%	4.63	0.95	
≥10	3.12%	99.33%	95.34%	4.63	0.98	
≥11	3.12%	99.60%	95.60%	7.72	0.97	
≥12	0.00%	99.60%	95.47%	0	1	

Pearson's correlation coefficient assessing the concurrent validity between the GHQ and WHODAS was low at 0.23. The concurrent validity assessed in the same manner with the GHQ and WHODAS number of disability days was also low at 0.18. Reliability was established in two ways. First, Cronbach's alpha was found measuring internal scale reliability at 0.99. Kappa coefficient for raters using both GHQ and MINI was found to be 0.11.

Item-level analysis (Table 3) found mean scores on each item ranged from 0.09 to 0.22. Pearson's item-total correlations were found to be moderate and ranged from 0.42 to 0.67. Scale reliability without each item assessed by Cronbach's alpha remained high with low variability, ranging from 0.80 to 0.82. Likelihood ratio positive for each item ranged from 1.01 to 6.30, proving that the majority of items are not discriminating CMD cases from non-cases well.

**Table 3:** Score on item of GHQ-12, Pearson item-total correlations, Cronbach's alphas, item likelihood ratio positive.

GHQ-12 items	Mean	SD	Pearson's item-total correlation	Cronbach's alpha, if item is deleted	LR+
Able to concentrate	0.09	0.29	0.67*	0.80	3.81
Lost sleep	0.14	0.35	0.56*	0.81	3.58
Playing useful part	0.06	0.23	0.56*	0.81	3.73
Capable of making decisions	0.12	0.33	0.42*	0.82	1.01
Felt under strain	0.22	0.41	0.59*	0.81	3.57
Could not overcome difficulties	0.09	0.28	0.63*	0.80	3.00
Enjoy daily activities	0.10	0.30	0.66*	0.80	3.10
Face up to problems	0.08	0.27	0.64*	0.80	4.03
Feeling unhappy and depressed	0.19	0.39	0.63*	0.80	3.79
Losing confidence in self	0.10	0.30	0.51*	0.81	3.49
Thinking of self as worthless	0.07	0.25	0.47*	0.81	6.30
Feeling reasonably happy	0.11	0.31	0.66*	0.80	3.73
				Cronbach's alpha	
Total GHQ score	1.36	2.17		0.82	

*P* values: * = 0.00

## 4 Discussion

We set out to establish the reliability and validity of the GHQ-12 in a community sample of men in India. While the GHQ-12 is highly internally consistent, a low cut-off score is best able to detect probable CMD in the study setting, with modest validity estimates.

However, when looking at the optimal cut off score of 2, the diagnostic odds ratio was found to be weak and much lower than the average found by a recent systematic review, which found an average DOR of 22.59 from 13 validation studies of the GHQ-12 (Ali et al., 2016). Item-level analysis proved that while the items had moderate correlation, only the item “thinking of self as worthless” had a moderate increase in correctly identifying CMD case. This item-analysis may account for the poor psychometric properties found.

Despite the recommended cut-off score of 8 from the WHO and the score of 6 from the previous GHQ-12 validation study in Goa (Patel et al., 2008) a cut-off score of 2 found in this study is not uncommon. Goldberg et al. demonstrated that the most common optimal cut-off score was 2/3 from a sample of 5428 patients interviewed in 15 centers from a WHO study. Across these 15 centers, optimal cut-off points varied from 1–2 to 6–7 (Goldberg et al., 1997). 17 other GHQ-12 validity studies showed a wide range of ideal cut-off scores from 0–1 to 5–6 (Goldberg et al., 1997, Goldberg et al., 1998). Finally, a validation of the GHQ-12 from a community sample of the general adult population in Korea found the optimal cut-off score for CMDs to be 1–2 (Kim et al., 2013), with an AUC of 0.632. The potential reasons for the large range in optimal threshold scores include differing prevalence rates of psychiatric disorders and comorbid diagnoses as well as cultural factors (Lewis and Araya, 1995, Ozdemir and Rezaki, 2007).

The GHQ-12 has been previously validated within primary care populations in Goa, and the optimal cut-off score was determined through the best balance between sensitivity and positive predictive value. Due to the nature of a resource-limited primary-care setting, the reduction of false positives is attractive (Patel et al., 2008). In a community survey, a lower cut-off score may be beneficial in order to reach all possible cases and reduce the number of false negatives. A validation study of the Tamil version of the GHQ-12 in the community noticed a differing predictive value for the version of the tool than had been found in previous hospital settings. Further, the prevalence of CMDs was found to be lower (John et al., 2006). John et al. note that tests such as the GHQ-12 may be less useful in a community survey where the prevalence of CMDs is low; however, they are indeed necessary and validation of such tools is required. For this reason, a lower cut-off score may need to be used when screening for CMDs in a community sample in Goa, India.

## 5 Limitations

As this study was not conducted primarily as a validation study for the GHQ-12, we were unable to provide inter-rater reliability as well as test re-test reliability; both important psychometric properties. Furthermore, although the sample was more inclusive and extensive than previous validation studies in Goa it excluded women, and our findings are therefore not generalizable. We also note that our gold standard MINI was not administered by a clinician as is recommended by Ali and colleagues; however, the MINI administered by a non-clinician was deemed acceptable both here and in the systematic review by Ali et al. (2016). It is also important to note that different scoring methods of the GHQ-12 have previously caused variation in psychometric properties and optimal cut-off scores depending on setting (Donath, 2001, Bakhla et al., 2013). Further studies comparing the validity of the GHQ-12 using varying scoring methods are suggested.

## 6 Conclusion

The GHQ-12 is useful in low-resource settings as a fairly efficient, effective screening tool for CMD, but at much lower cut-offs if used in community settings. Contrary to the view held by John and colleagues that the GHQ-12 may be less useful in non-primary care settings (John et al., 2006), we think part of the reasons for the low levels of identification and treatment for CMDs in under-resourced community settings is because of the high cut-off recommended by WHO (these patients would otherwise be missed if the WHO criteria were used). However, reaching this conclusion (that would contribute to prompt interventions and thus reduction in the treatment gap) would require further investigations in different under-resourced settings using appropriate and rigorous psychometric study designs.

## Funding

This work was supported by the Wellcome Trust Research Training Fellowship to Abhijit Nadkarni [grant number WT093897MA].

## Conflict of interest statement

None declared.

## References

[bib0005] Adewuya A.O. (2006). Validation of the Edinburgh Postnatal Depression Scale as a screening tool for depression in late pregnancy among Nigerian women. J. Psychosom. Obstet. Gynaecol..

[bib0010] Ali G.C., Ryan G., De Silva M.J. (2016). Validated screening tools for common mental disorders in low and middle income countries: a systematic review. PLoS One.

[bib0015] Bakhla A.K. (2013). Internal consistency and factor structure of 12-item general health questionnaire in visually impaired students. Ind. Psychiatry J..

[bib0020] Carey K.B., Carey M.P., Chandra P.S. (2003). Psychometric evaluation of the alcohol use disorders identification test and short drug abuse screening test with psychiatric patients in India. J. Clin. Psychiatry.

[bib0025] Chandramouli C., India (2011). Office of the Registrar General & Census Commissioner., Census of India, 2011. Series 1, India. Paper..

[bib0030] Donath S. (2001). The validity of the 12-item General Health Questionnaire in Australia: a comparison between three scoring methods. Aust. N. Z. J. Psychiatry.

[bib0035] Fluss R., Faraggi D., Reiser B. (2005). Estimation of the Youden Index and its associated cutoff point. Biom. J..

[bib0040] Goldberg D. (1979). GHQ and psychiatric case. Br J Psychiatry.

[bib0045] Goldberg D.P., Huxley P. (1992). Common Mental Disorders: A Bio-social Model.

[bib0050] Goldberg D.P. (1997). The validity of two versions of the GHQ in the WHO study of mental illness in general health care. Psychol. Med..

[bib0055] Goldberg D.P., Oldehinkel T., Ormel J. (1998). Why GHQ threshold varies from one place to another. Psychol. Med..

[bib0060] John S. (2006). Validation and usefulness of the Tamil version of the GHQ-12 in the community. Br. J. Community Nurs..

[bib0065] Kessler R.C. (2009). The global burden of mental disorders: an update from the WHO World Mental Health (WMH) surveys. Epidemiol. Psichiatr. Soc..

[bib0070] Kim Y.J. (2013). The 12-Item General Health Questionnaire as an effective mental health screening tool for general Korean adult population. Psychiatry Investig..

[bib0075] Kirmayer L.J. (2001). Cultural variations in the clinical presentation of depression and anxiety: implications for diagnosis and treatment. J. Clin. Psychiatry.

[bib0080] Lecrubier Y.S., Weiller D.V., Amorim E., Bonora P., Harnett Sheehan I., Janavs K., Dunbar J., G.C (1997). The Mini International Neuropsychiatric Interview (MINI). A short diagnostic structured interview: reliability and validity according to the CIDI. Eur. Psychiatry.

[bib0085] Lewis G., Araya R.I. (1995). Is the General Health Questionnaire (12 item) a culturally biased measure of psychiatric disorder?. Soc. Psychiatry Psychiatr. Epidemiol..

[bib0090] Ozdemir H., Rezaki M. (2007). General Health Questionnaire-12 for the detection of depression. Turk. Psikiyatri. Derg..

[bib0095] Patel V., Prince M. (2006). Maternal psychological morbidity and low birth weight in India. Br. J. Psychiatry.

[bib0100] Patel V. (2008). Detecting common mental disorders in primary care in India: a comparison of five screening questionnaires. Psychol. Med..

[bib0105] Pillai A. (2013). Patterns of alcohol use, their correlates, and impact in male drinkers: a population-based survey from Goa, India. Soc. Psychiatry Psychiatr. Epidemiol..

[bib0110] Schmitz N., Kruse J., Tress W. (1999). Psychometric properties of the General Health Questionnaire (GHQ-12) in a German primary care sample. Acta Psychiatr. Scand..

[bib0115] Steel Z. (2014). The global prevalence of common mental disorders: a systematic review and meta-analysis 1980–2013. Int. J. Epidemiol..

[bib0120] Whiteford H.A. (2013). Global burden of disease attributable to mental and substance use disorders: findings from the Global Burden of Disease Study 2010. Lancet.

